# Multisystem infection with cavernous sinus thrombosis in seropositive rheumatoid arthritis: a clinico-radiological case report

**DOI:** 10.1097/MS9.0000000000005238

**Published:** 2026-08-12

**Authors:** Kumari Madhulata, Uzma Jawaid, Rishi Tarafdar, Shreya Khandelwal

**Affiliations:** a Department of General Medicine, MGM Medical College, Kishanganj, India; b Department of Ophthalmology, MGM Medical College, Kishanganj, India

**Keywords:** case report, cavernous sinus thrombosis, cerebellar abscess, painful ophthalmoplegia, rheumatoid arthritis, septic emboli, superior ophthalmic vein thrombosis

## Abstract

**Introduction:**

Rheumatoid arthritis (RA) is a chronic systemic autoimmune disease primarily affecting synovial joints, but it may also manifest with extra-articular involvement, including neurological and ophthalmic complications. Cavernous sinus thrombosis (CST) is a rare yet life-threatening condition, most often secondary to infection, characterized by painful ophthalmoplegia, proptosis, and cranial nerve dysfunction. The coexistence of RA, disseminated infection, and CST is exceedingly uncommon and presents significant diagnostic and therapeutic challenges. This case highlights the importance of early recognition of atypical ophthalmic and neurological manifestations in patients with autoimmune disease.

**Case presentation:**

A diabetic patient with a 6-month history of progressive symmetrical polyarthralgia, joint swelling, deformities, and upper motor neuron signs presented with acute right-sided painful ophthalmoplegia and proptosis. Clinical evaluation revealed seropositive RA with elevated inflammatory markers and leukocytosis. A neuro-ophthalmic examination demonstrated chemosis, sixth cranial nerve palsy, and raised intraocular pressure. Magnetic resonance imaging of the brain and orbits revealed bilateral superior ophthalmic vein thrombosis, cavernous sinus involvement, multiple intracranial and deep facial space abscesses, and cervicomedullary junction compression, consistent with disseminated infection and septic emboli.

**Clinical discussion:**

This case underscores the diagnostic complexity arising from overlapping autoimmune and infectious processes. Immune dysregulation associated with RA and diabetes likely predisposed the patient to disseminated infection and secondary venous thrombosis. Early ophthalmic signs served as critical diagnostic clues, while magnetic resonance imaging was instrumental in identifying the extent of multisystem involvement. Prompt initiation of broad-spectrum antibiotics, systemic corticosteroids, anticoagulation, and multidisciplinary management resulted in significant clinical improvement.

**Conclusion:**

This case illustrates a rare and severe multisystem presentation of RA, complicated by disseminated infection and CST. It emphasizes the need for heightened clinical vigilance, early neuroimaging, and an integrated therapeutic approach when patients with autoimmune disease present with painful ophthalmoplegia and neurological deficits to prevent potentially fatal outcomes.

## Introduction

Rheumatoid arthritis (RA) is a chronic, systemic autoimmune inflammatory disease defined by persistent synovitis, symmetrical polyarthritis, and progressive joint destruction. While articular manifestations are the hallmark of the disease, RA is increasingly recognized as a multisystem disorder with a broad spectrum of extra-articular complications that contribute substantially to morbidity and mortality^[^1^]^. Extra-articular manifestations – including cutaneous, pulmonary, cardiovascular, ocular, and neurologic involvement – occur more frequently in patients with seropositive, aggressive, or long-standing disease and are associated with poorer functional status and reduced survival^[^2^]^. The presence of metabolic comorbidities, such as diabetes mellitus, further magnifies susceptibility to infection and accelerates systemic complications, making clinical trajectories more unpredictable^[^3^]^.

HIGHLIGHTS

- RA may cause life-threatening infections, venous thrombosis, and spine instability.
- Painful ophthalmoplegia warrants an urgent evaluation for CST.
- Facial infections in the danger triangle can spread intracranially without a source.
- Septic spread caused brain abscess, lung nodules, effusions, and thrombosis.
- Atlanto-axial pannus and infection complicated neurological assessment and care.

Pulmonary involvement in RA is well-documented and may encompass pleural disease, interstitial lung disease, rheumatoid nodules, and airway inflammation. Although pleural effusions and cavitating pulmonary nodules are relatively uncommon, they are recognized complications that may reflect underlying serositis, rheumatoid nodulosis, or superimposed infection^[^4^]^. Neurologic manifestations of RA are rare but potentially devastating. They most often result from articular destruction – such as atlanto-axial instability leading to cervicomedullary compression – or from small-vessel vasculitis affecting peripheral nerves, meningeal inflammation, or ischemic neuropathies. When RA involves the cervical spine, erosive changes and ligamentous laxity at the craniovertebral junction may produce myelopathy or life-threatening brainstem compression^[^5^]^.

Ocular involvement in RA typically presents as keratoconjunctivitis sicca, episcleritis, or scleritis; these conditions are well-recognized and often readily managed^[^6^]^. In contrast, painful ophthalmoplegia, chemosis, and proptosis are unusual in RA and should prompt urgent evaluation for orbital apex or cavernous sinus pathology. Cavernous sinus thrombosis (CST), particularly in its septic form, represents a rare but devastating neurovascular complication. Infection originating within the so-called “danger triangle” of the face can spread to the cavernous sinus through valveless venous channels, sometimes even in the absence of an obvious dental or sinonasal focus^[^7^]^. CST classically presents with painful ophthalmoplegia, cranial nerve deficits (most commonly abducens nerve palsy), chemosis, and proptosis and requires a high index of suspicion because early signs may be subtle and easily misattributed to orbital inflammation or disease flares^[^8^]^.

The confluence of disseminated infection, venous thrombosis, and autoimmune inflammatory disease is exceptionally uncommon. Our patient demonstrated a unique constellation of superior ophthalmic vein and CST presenting as painful ophthalmoplegia, together with cerebellar abscess, cavitating pulmonary nodules with bilateral pleural effusions, polyarticular fluid collections, and erosive atlanto-axial disease resulting in cervicomedullary compression. To our knowledge, the simultaneous occurrence of these features in a single seropositive RA patient has not previously been reported. We present this case to emphasize the importance of considering septic cavernous sinus involvement when patients with RA develop acute painful ophthalmoplegia and to highlight the need for prompt imaging and coordinated multidisciplinary care to avert irreversible neurological sequelae. This case has been prepared in accordance with established CARE case-report guidelines^[^9^]^. No artificial intelligence tools were used in the preparation, interpretation, or writing of this manuscript, in accordance with the TITAN guidelines^[^10^]^.

## Case presentation and diagnosis

A middle-aged man with diabetes mellitus presented with a 6-month history of progressive, symmetrical inflammatory polyarthritis characterized by diffuse body pain, swelling and deformity of both wrists, restricted joint mobility, and increasing difficulty in standing and walking. He also reported stiffness, cracking sensations in multiple joints, and generalized paresthesias. There was no history of fever, trauma, recent surgery, sinus complaints, dental infection, visual loss, or prior immunosuppressive therapy. He had a history of cannabis abuse for 6 years, which he discontinued 2 years earlier. On admission, he was afebrile, hemodynamically stable, and oriented, with a random blood glucose level of 311 mg/dL. General physical examination was unremarkable; however, musculoskeletal examination demonstrated chronic deforming polyarthritis. Neurological examination revealed increased tone, exaggerated deep tendon reflexes, sustained clonus, bilateral extensor plantar responses, and mildly reduced power (4/5) in all four limbs, suggestive of upper motor neuron involvement.

**Figure 1.**
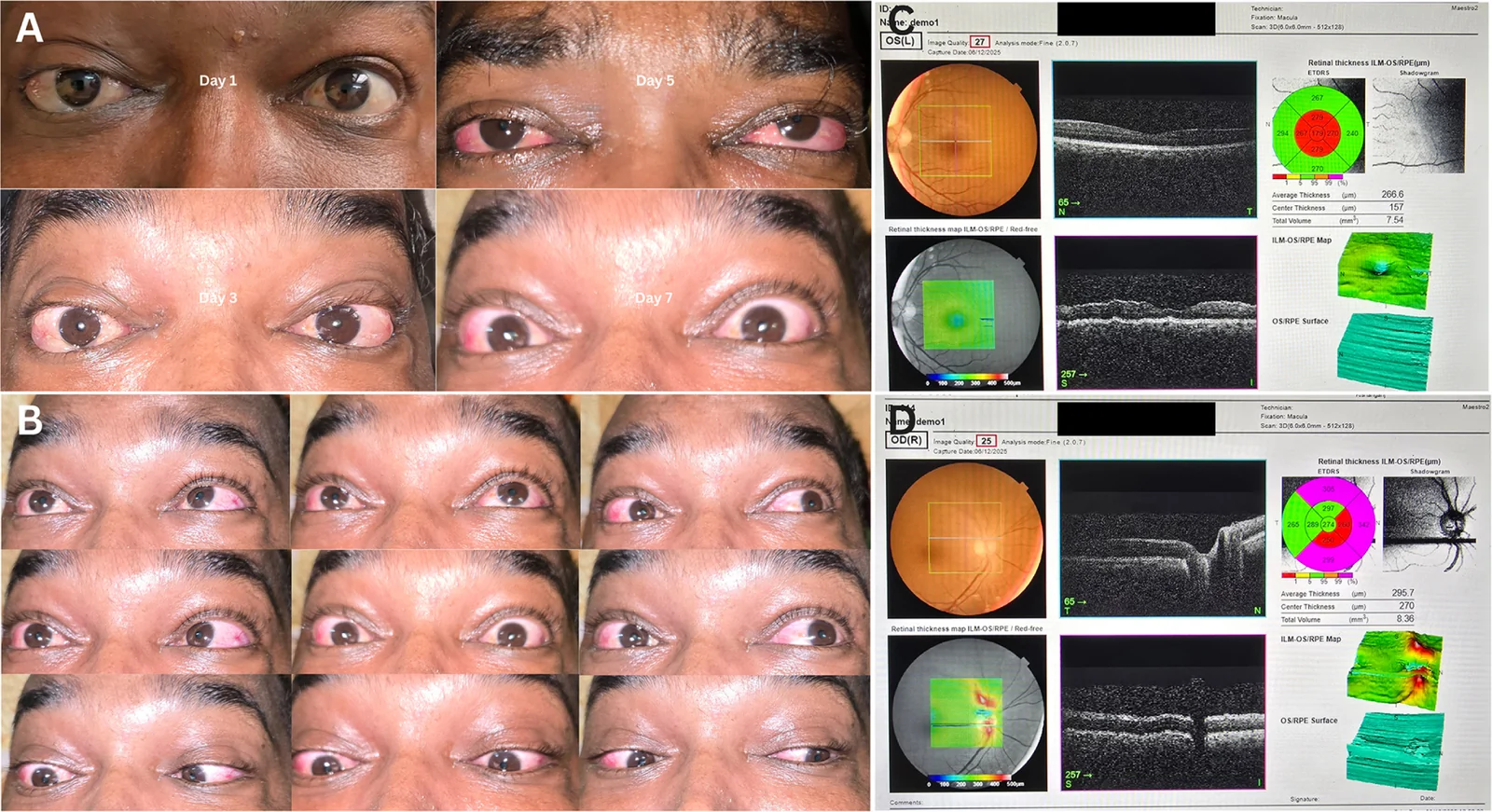
(A) Clinical course of chemosis (Day 1–Day 7). (B) Nine-gaze examination showing limitation of abduction in the right eye consistent with sixth cranial nerve palsy at presentation. (C) Optical coherence tomography (OCT) of the Left eye macula. (D) Optical coherence tomography (OCT) of the Right eye macula.

During hospitalization, his course evolved significantly. On the third hospital day, he developed acute right periorbital swelling, followed by painful restriction of eye movements. Serial external photographs demonstrated the evolution of chemosis (Fig. 1A). By Day 5, ophthalmic examination revealed diffuse eyelid edema, marked conjunctival chemosis, mild proptosis, and limitation of abduction consistent with right sixth cranial nerve palsy (Fig. 1 B); intraocular pressure was digitally elevated, while pupils and fundus were normal. Initially unilateral, the chemosis progressed and subsequently became bilateral, reaching maximal severity on Day 5 before gradually resolving over the ensuing week, with preservation of vision. Optical coherence tomography showed central macular thinning in both eyes (Fig. 1C and D). Systemic examination otherwise remained unremarkable.

Laboratory investigations demonstrated leukocytosis (20 510/µL), normocytic anemia, elevated CRP, hypoalbuminemia, and good renal and hepatic function. Serology revealed a reactive rheumatoid factor (32 IU/mL) with negative antinuclear antibodies. Glycemic indices confirmed suboptimal control. Given the emergence of painful ophthalmoplegia in the context of systemic inflammatory disease and sepsis, urgent magnetic resonance imaging of the brain, orbits, head, and neck was obtained.

**Figure 2.**
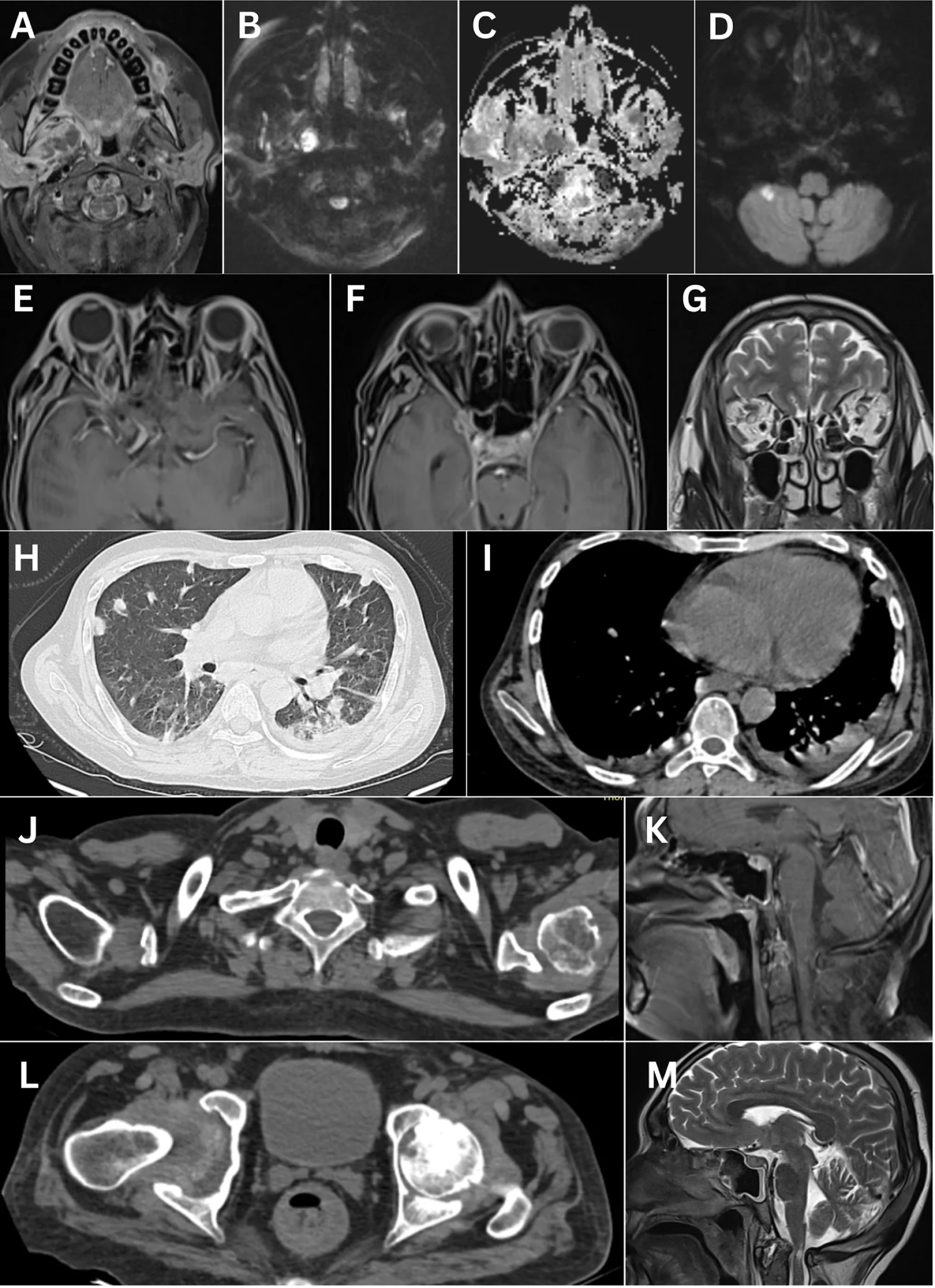
(A–G) MRI brain and orbit: septic spread and cavernous sinus involvement. (H–M) Systemic dissemination: thorax, pelvis, shoulders, and craniovertebral junction. (A) Axial T1-weighted fat-suppressed post-contrast image shows peripheral ring-enhancing collection in the right masticator space involving the pterygoid muscles, consistent with abscess. (B) Similar enhancing lesion is seen in the left maxillary space extending to the retromolar trigone, suggesting bilateral deep facial space infection. (C) Diffusion-weighted sequences demonstrate diffusion restriction in the bilateral masticator spaces, more pronounced on the right. (D) Diffusion-weighted imaging with corresponding ADC map shows focal diffusion restriction in the right anterior cerebellar hemisphere, compatible with abscess. (E) Axial post-contrast T1-weighted fat-suppressed images reveal intraluminal filling defects within both superior ophthalmic veins, consistent with superior ophthalmic vein thrombosis. (F) Post-contrast images demonstrate abnormal thickening and enhancement of the cavernous sinus, compatible with cavernous sinus thrombosis. (G) Coronal T2-weighted orbital sequences show dilated superior ophthalmic veins with loss of normal flow voids and hyperintense signal, indicating orbital venous congestion. (H) CT thorax (lung window) shows multiple soft-tissue density nodules, several with central cavitation, suggestive of septic emboli. (I) CT thorax (soft tissue window) reveals bilateral pleural effusions. (J) Non-contrast CT pelvis demonstrates fluid collections in both hip joints (right > left) with articular surface irregularities. (K) Axial T1-weighted fat-suppressed MRI shows abnormal enhancement at the craniovertebral junction. (L) Similar inflammatory fluid and synovial changes are seen in both shoulder joints. (M) Sagittal T2-weighted MRI shows fluid extending across the craniovertebral junction with foramen magnum narrowing, spinal cord compression, and cord edema.

MRI demonstrated extensive, anatomically contiguous disease (Fig. 2A–G). Post-contrast sequences showed peripherally enhancing collections within the right masticator space, measuring approximately 2.8 × 1.9 cm, extending along intermuscular planes and involving the pterygoid and masseter muscles, with associated inflammatory edema. Diffusion-weighted imaging revealed bilateral masticator space diffusion restriction, more pronounced on the right (Fig. 2C), consistent with deep facial space abscesses.

A focal lesion in the right anterior cerebellar hemisphere, measuring ~1.2 cm, exhibited marked diffusion restriction with low ADC values and peripheral ring enhancement (Fig. 2D), compatible with cerebellar abscess formation. In addition, there was abnormal dural thickening and enhancement along both cavernous sinuses (Fig. 2F), with intraluminal filling defects and non-visualization of the right superior ophthalmic vein on post-contrast sequences (Fig. 2E). These findings, together with dilated superior ophthalmic veins, orbital venous congestion, and proptosis on coronal T2-weighted orbital imaging (Fig. 2G), indicated bilateral superior ophthalmic vein thrombosis with CST. Clinically, this correlated with painful ophthalmoplegia and right abducens nerve palsy (Fig. 1B), accompanied by progressive chemosis (Fig. 1A).

Systemic dissemination was further evident on cross-sectional imaging (Fig. 2H–M). CT thorax demonstrated multiple randomly distributed pulmonary nodules, several showing central cavitation (Fig. 2H), along with bilateral pleural effusions (Fig. 2I), findings highly suggestive of septic embolization. Musculoskeletal imaging revealed synovial fluid collections and articular surface irregularities involving both hip joints (right > left) (Fig. 2J) and bilateral shoulders (Fig. 2L), raising concern for inflammatory synovitis with possible superimposed infection.

Cervical spine imaging showed advanced atlanto-axial arthropathy with pannus formation, posterior dens subluxation, narrowing of the foramen magnum, and T2 hyperintensity within the cervicomedullary cord, consistent with myelomalacia (Fig. 2K and M), correlating with the patient’s upper motor neuron signs.

Integrating clinical, laboratory, and radiologic findings, a final diagnosis of seropositive RA with atlanto-axial instability complicated by disseminated infection and septic emboli was established, with secondary cavernous sinus and orbital apex involvement resulting in painful ophthalmoplegia and superior ophthalmic vein thrombosis.

The patient was managed with broad-spectrum intravenous antibiotics, therapeutic anticoagulation, carefully supervised systemic corticosteroids, aggressive metabolic control, and multidisciplinary supportive care. Over the subsequent 5–7 days, chemosis progressively resolved (Fig. 1A), ocular motility and proptosis improved, and vision remained preserved (6/9 in the right eye and 6/6 in the left eye, with stable macular architecture on optical coherence tomography; Fig. 1C–D). Inflammatory markers normalized, and the patient demonstrated gradual neurological stabilization.

This case underscores the critical importance of recognizing painful ophthalmoplegia as an early sentinel sign of cavernous sinus involvement, particularly in patients with underlying autoimmune disease. It also highlights the indispensable role of prompt multimodal imaging and coordinated, aggressive management in preventing catastrophic neurological and visual sequelae.

## Differential diagnosis

The combination of chemosis, painful ophthalmoplegia, and sixth cranial nerve palsy initially raised a broad differential that included CST, orbital cellulitis, idiopathic orbital inflammatory disease, carotid-cavernous fistula, thyroid-associated orbitopathy, and systemic vasculitic disorders. In this patient, the rapid bilateral progression, abducens nerve involvement, and marked venous congestion strongly favored cavernous sinus pathology. The absence of dental or sinus disease, lack of pulsatile proptosis or bruit, normal thyroid profile, negative autoimmune serology, and the presence of disseminated infection argued against these alternative diagnoses. MRI demonstrating superior ophthalmic vein thrombosis with cavernous sinus involvement ultimately confirmed septic CST as the unifying explanation for the ophthalmic findings.

## Discussion

This case illustrates the breadth and complexity of systemic disease in RA and highlights how autoimmune inflammation, occult infection, thrombosis, and structural degeneration may converge in a single, rapidly evolving clinical presentation. The patient initially exhibited features of chronic, symmetrical inflammatory polyarthritis with upper motor neuron signs, findings consistent with long-standing seropositive disease complicated by cervical spine involvement^[^11^]^. Diabetes mellitus – poorly controlled at presentation – likely compounded host vulnerability, impairing neutrophil function and facilitating deeper infectious spread^[^12^]^.

The clinical inflection point occurred with the abrupt onset of painful ophthalmoplegia and evolving chemosis. Whereas ocular complaints in RA are usually attributed to anterior segment disease, the presence of sixth-nerve palsy, venous congestion, and bilateral progression signaled cavernous sinus pathology rather than primary ocular inflammation^[^13^]^. Notably, there was no antecedent dental or sinonasal focus. Nonetheless, infection arising within the “danger triangle” of the face may propagate retrogradely through valveless venous channels to the cavernous sinus, where inflammation, venous stasis, and hypercoagulability intersect^[^14^]^. Magnetic resonance imaging confirmed superior ophthalmic vein thrombosis with cavernous sinus enhancement and orbital venous congestion, providing a unifying explanation for the patient’s neuro-ophthalmic presentation^[^15^]^.

The accompanying pattern of pathology was strikingly systemic. Deep facial space abscesses, cerebellar abscesses, mastoid involvement, cavitating pulmonary nodules, pleural effusions, and polyarticular joint effusions together suggested hematogenous dissemination with septic embolization^[^16^]^. The imaging narrative – from head and neck to chest and spine – revealed a contiguous pathophysiological sequence: local infection tracking venously, entering the systemic circulation, and seeding distant structures. In this context, broad-spectrum antimicrobial therapy and early source control became paramount^[^17^]^.

Superimposed cervical spine disease added substantial complexity. Atlanto-axial pannus with posterior dens subluxation and narrowing at the foramen magnum accounted for the long-standing pyramidal signs on examination and created a precarious risk of cervicomedullary compression. Cervical pathology in RA is often radiographically silent until neurologic compromise occurs; in this case, the coexistence of instability and systemic infection required careful balancing of immobilization, neurological surveillance, and delayed surgical consideration^[^18^]^.

The presence of large-joint effusions in the hips and shoulders further complicated interpretation. In patients with RA, distinguishing inflammatory synovitis from septic arthritis remains clinically challenging, as both produce pain, swelling, and elevated inflammatory markers. In the setting of disseminated infection, prudence dictates that effusions be considered septic until demonstrated otherwise. Improvement with antimicrobial therapy supported an inflammatory-infective overlap rather than an isolated autoimmune exacerbation^[^19^]^.

That the patient ultimately improved underscores the value of timely, coordinated intervention. Early neuroimaging, rapid initiation of empiric antibiotics, cautious anticoagulation, and judicious corticosteroid use – introduced only after the infection was addressed – were central to preserving vision and stabilizing neurological function^[^20^]^. The multidisciplinary collaboration among rheumatology, neurology, ophthalmology, infectious disease, radiology, and critical care exemplifies best practice in complex multisystem disease.

Several lessons emerge. RA must be understood as a systemic immunoinflammatory disorder in which extra-articular complications may define prognosis^[^21^]^. Painful ophthalmoplegia in this population constitutes a red-flag event, demanding urgent evaluation for cavernous sinus or orbital venous pathology^[^22^]^. Disseminated infection, particularly in the presence of diabetes, can mimic an autoimmune flare and must be excluded before immunosuppression is escalated^[^23^]^. Finally, vigilance for cervical spine instability remains essential in long-standing disease, particularly when upper motor neuron signs evolve^[^24^]^.

In summary, this case presents an unusual and instructive convergence of septic dissemination, cavernous sinus and superior ophthalmic vein thrombosis, cerebellar and pulmonary embolic involvement, and rheumatoid cervical spine disease. The outcome highlights the importance of early suspicion, comprehensive radiologic assessment, and disciplined multidisciplinary care in preventing irreversible neurological and visual morbidity^[^25^]^. It serves as a reminder that seemingly routine complaints in patients with RA may herald profound systemic pathology requiring a prompt, integrated clinical response.

## Conclusion

RA is a systemic autoimmune disorder that may rarely be complicated by severe multisystem involvement, including neurological and ophthalmic manifestations. Painful ophthalmoplegia should be regarded as an important clinical warning sign, warranting urgent evaluation for cavernous sinus pathology, even in the absence of overt sinonasal infection. This case highlights the value of early neuroimaging in establishing the diagnosis and delineating the extent of disease. Timely initiation of appropriate antimicrobial therapy, judicious use of corticosteroids, and anticoagulation, in conjunction with multidisciplinary care, may result in favorable clinical outcomes. Clinicians should maintain a high index of suspicion for infectious and thrombotic complications in patients with autoimmune disease and diabetes who present with atypical neurological or ocular symptoms.

## Methods

This case report has been prepared and reported in accordance with the CARE 2023 guidelines for medical case reports.

## Data Availability

Not applicable.
